# Occupational associations of preserved ratio impaired spirometry (PRISm) in a population-based study

**DOI:** 10.3389/fpubh.2026.1921126

**Published:** 2026-09-22

**Authors:** Denis Vinnikov, Irina Mukatova, Zhanar Urazalina, Zhangir Dairuly, Aizhan Raushanova, Zhanna Romanova

**Affiliations:** 1 Environmental Health Lab, Al-Farabi Kazakh National University, Almaty, Kazakhstan; 2 Occupational Health Risks Lab, Peoples’ Friendship University of Russia (RUDN University), Moscow, Russia; 3 Department of Internal Diseases with Courses of Nephrology, Hematology, Allergology, and Immunology, Astana Medical University, Astana, Kazakhstan; 4 Department of Emergency Medicine, Semey Medical University, Semey, Kazakhstan; 5 Department of Epidemiology, Biostatistics and Evidence-Based Medicine, Al-Farabi Kazakh National University, Almaty, Kazakhstan

**Keywords:** job-exposure matrix, Kazakhstan, occupational, population-based, work-related

## Abstract

**Introduction:**

Preserved ratio impaired spirometry (PRISm) has never been characterized in Kazakhstan, and risk factors for this condition remain poorly described. Given that smoking was associated with PRISm, other sources of air pollution, including occupational exposures, surprisingly remained outside the scope of many studies. We, therefore, aimed to compute the prevalence and test the association of exposure to airborne pollutants in the workplace with PRISm in a cross-sectional study.

**Methods:**

Subjects (*N* = 2,275, median age 41 (interquartile range (IQR) 29; 56) years, 54% men) were recruited from the general population in Aktobe, Karaganda, and Kostanay. They completed the questionnaire and underwent spirometry to measure z-scores of forced expiratory volume in 1 s (FEV1) and forced vital capacity (FVC). Exposures in the workplace were assessed based on jobs held throughout a lifetime using a job-exposure matrix (JEM), and the association with PRISm was tested in adjusted logistic regression.

**Results:**

PRISm was confirmed in 12% of participants (95% confidence interval (CI) 10.7; 13.3), with a higher prevalence observed in women (14, 95% CI 11.7; 15.6) compared to men (10, 95% CI 8.8; 12.2). Occupational airborne exposures, including vapor, gas, dust and fume (VGDF), were not associated with PRISm, either as ever-exposure or as cumulative exposure in years. Body mass index [adjusted odds ratio (OR) 1.05 (95% CI 1.02; 1.07)] and the Charlson comorbidity index [adjusted OR 1.28 (95% CI 1.07; 1.54)] were significantly associated with the outcome, after adjusting for sex, smoking, regular exercise, age, and VGDF exposure.

**Conclusion:**

In the current study, occupational airborne chemical exposures, classified using a JEM, showed a non-significant association with PRISm. However, further research with larger sample sizes is needed.

## 1 Introduction

Preserved ratio impaired spirometry (PRISm) has recently gained much attention because gaps exist in understanding how to manage subjects with no obvious clinical signs and no obstruction defined as a reduced forced expiratory volume in 1 s (FEV1) to forced vital capacity (FVC) ratio, but with some reduction in FEV1. This phenomenon has only recently become widely reported, but existing evidence from biobank studies and population-based reports tends to characterize this as a common condition (1–3), sometimes even more prevalent than chronic obstructive pulmonary disease (COPD). Limited evidence from such studies also concluded that PRISm may progress to COPD (3, 4), more often in subjects with risk factors, including cigarette smoking, air pollution, or occupational exposure to dust; however, data are yet inconsistent and further studies are needed (1).

Studies elsewhere reported contradictory findings on PRISm prevalence in the general population (2, 3, 5), and such inconsistency may arise from heterogeneity in definition, given that PRISm may be defined both using the lower limit of normality (LLN) for FEV1/FVC and FEV1 or, alternatively, a reduction below 80% of the predicted value for FEV1 with normal FEV1/FVC. Overall, its prevalence may widely range from 5.5% (age-standardized) in the China Pulmonary Health Study (5) to 11% in UK Biobank (2) and even 12.3% in COPDGene (3), but more important is its potential association with clinical outcomes and prognosis. Emerging evidence concludes that some association exists, but it also leaves clinicians puzzled about how to manage these patients, provide counseling, and determine whether further monitoring is needed. At present, no clear consensus on these issues can be considered conventional, but its relatively high prevalence necessitates further research.

At present, PRISm is considered to be strongly associated with increased body mass index (BMI), heavier comorbidity, age, female sex, cigarette smoking, and even ambient air pollution (1, 6–8). Although cigarette smoking and air pollution are recognized as PRISm risk factors, there are surprisingly few studies focusing specifically on airborne occupational exposures. Furthermore, epidemiological studies on PRISm only briefly address workplace exposures only briefly. In addition, since PRISm often progresses to COPD (1, 4, 9) and COPD has long been associated with occupational exposures (10, 11), ascertaining the association between vapor, gas, dust, and fume (VGDF) and PRISm is worthwhile and warrants further exploration. Therefore, this population-based study aimed to compute the prevalence and to test the association between exposure to airborne pollutants in the workplace and PRISm.

## 2 Methods

### 2.1 Study design

This population-based study was planned and completed in three cities of the Republic of Kazakhstan, including Aktobe, Kostanay, and Karaganda. The study was approved by the Committee on Bioethics of al-Farabi Kazakh National University (approval IRB-A387, dated 17.03.2022), and all subjects who agreed to participate provided informed written consent. Data collection was initiated in February 2025 and ended on 31 May 2025. All subjects in these three cities were recruited by nurses by telephone from the list of enrolled population for one major outpatient facility (polyclinic) in each city. These polyclinics usually serve up to 30,000 people from the surrounding neighborhood of all ages, including newborns and older adults. One polyclinic in each city was specifically chosen for its location in the city center. The original nationwide study was completed in five cities (12), but only the three cities named above were selected for the current presentation because the occupational histories of the participants were complete in those cities (Figure 1). Participants were selected from the enlisted population of local polyclinics using random numbers as described (12), and in case a person failed to attend, the next person on the list was invited. After being informed and agreeing to participate, study participants arrived at the polyclinic on the morning of the appointment and were interviewed by a trained medical doctor. Spirometry was conducted right after the interview by either a trained nurse or a medical doctor. Sample size calculation was completed at a preceding stage of a larger study (12), and at least 2000 participants were needed to provide sufficient power.

**Figure 1:**
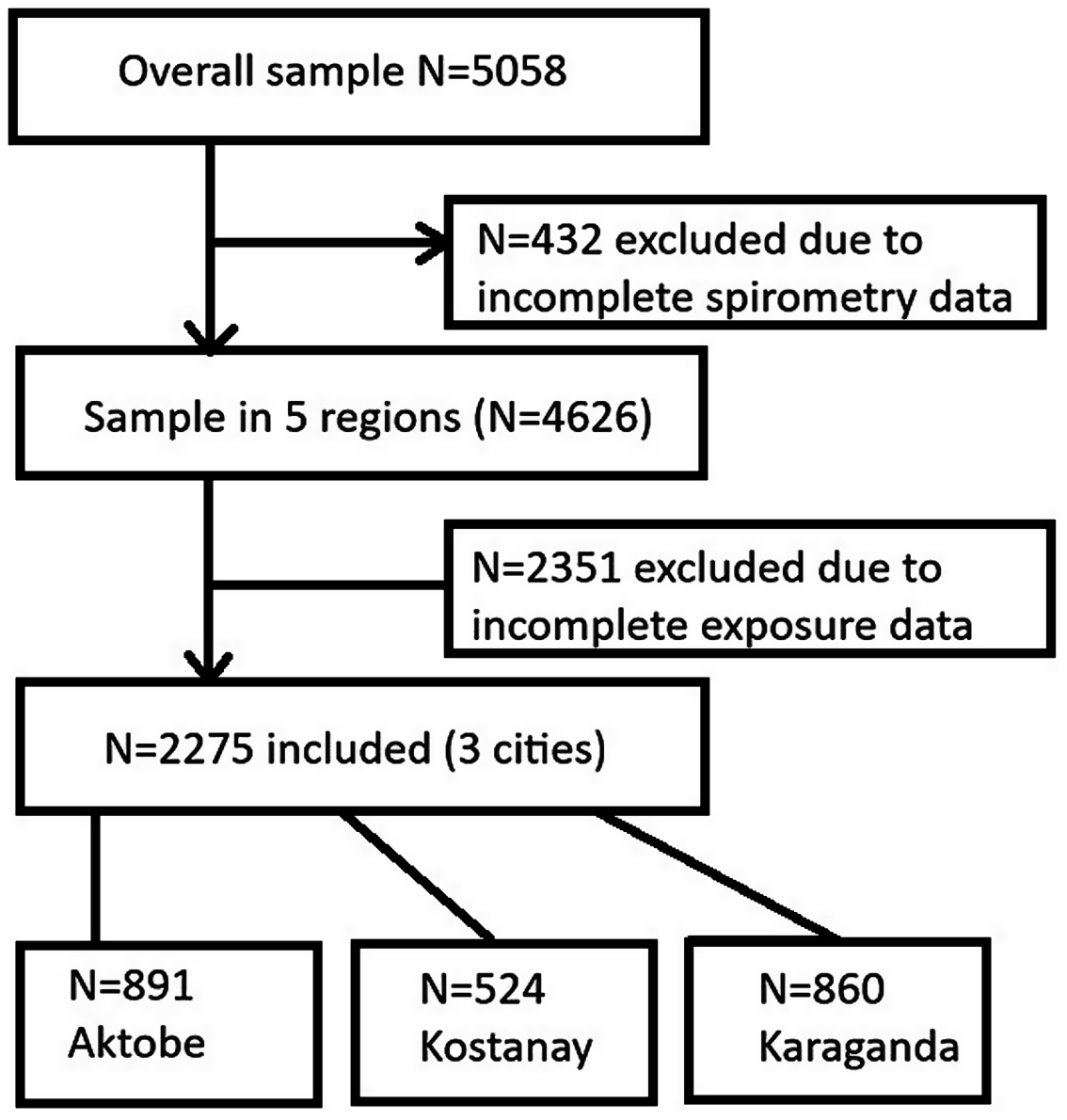
Flow diagram illustrating participants’ recruitment.

### 2.2 Questionnaire

We adapted the questionnaire we had reported before in similar population-based studies of respiratory outcomes and occupational exposures (12). Participants chose either Russian or Kazakh versions of identical questionnaires, and a trained nurse or a doctor assisted in filling out the questionnaire to ensure that there were no unanswered questions. The questionnaire was designed to include basic demographic information, including date of birth, place of residence, sex, cumulative family income, and highest attained education, followed by a detailed lifestyle and exposure section. We classified each participant as a never, former, or current cigarette smoker using a set of four questions, also clarifying the number of cigarettes smoked per day and years of smoking. We then asked about electronic cigarette use and its frequency, vaping and its frequency, waterpipe use and even exposure to secondhand smoke in the workplace or at home.

A detailed occupational history was also collected. All jobs held for more than a year in a lifetime were first recorded along with the years in service and place of work. We then used a job-exposure matrix (JEM) to convert jobs into occupational airborne chemical exposures. We chose the airborne chemical exposure (ACE) JEM (13) because it not only allowed us to derive a composite measure of a binary variable of exposure but also allowed us to classify workplace exposure specifically for each of eleven domains, including vapor, gas, dust, fume, bio-dust, mineral dust, diesel, fiber, mist, asbestos, and metal. In population-based studies, converting job data into exposures could provide more precise information on the actual chemical exposure in the workplace (14) than asking participants what exactly they had been exposed to. Therefore, in addition to one composite binary variable of exposure to VGDF, we also used eleven other binary variables of specific exposures (15). Years of exposure to VGDF were also recorded.

Regular exercise three or more times a week was also classified in a questionnaire. Clinical signs of respiratory disease were quantified with a COPD Assessment Test (CAT), which contained eight questions, each grading the severity of symptoms from 0 to 5, yielding a total score of 0 to 40. A score above 10 was usually considered “more symptoms,” but in our study, we treated the CAT score as a continuous variable. Finally, dyspnea severity was assessed with the modified Medical Research Council (mMRC) tool, grading it from 0 to 4. Height and weight were measured before spirometry. We computed and analyzed BMI in kg/m2. We used the Charlson Comorbidity Index (CCI) to estimate comorbidity burden in our patients. It was developed to compute 10-year survival in patients based on a combination of 19 chronic diseases and conditions. We transformed self-reported chronic diagnoses to the scores of CCI and produced the overall CCI for each patient.

### 2.3 Spirometry

Immediately after the completion of the questionnaire, subjects were asked to undergo office spirometry using a MAS-2PC (Belintelmed LLC, Belarus) portable spirometer attached to a notebook and running under IntelSpiro software integrating European Respiratory Society/American Thoracic Society (ERS/ATS) technical (16) and interpretative (17) protocols. The spirometer we used had an accuracy of ±3%, compliant with the existing standard for spirometers. We aimed to obtain at least three acceptable and repeatable curves graded A or B both for Δforced expiratory volume in 1 s (FEV1) and Δforced vital capacity (FVC) in a forced maneuver. For each of these, along with FEV1/FVC, we computed z-scores (N standard deviations from the predicted value) based on the individual predicted values using Global Lung Function Initiative (GLI)-2012 reference values. The lower limit of normality (LLN, z-score lower than −1.645) was used to discriminate between normal and abnormal values for each volume or ratio. Bronchodilation with four doses of salbutamol via a spacer was done in all subjects with FEV1/FVC below the LLN. Given that in PRISm FEV1/FVC is initially normal, we did not consider bronchodilation in PRISm subjects. PRISm was considered when FEV1/FVC was above LLN, but FEV1 was below LLN. This group also comprised subjects with suspected restriction (those having normal FEV1/FVC but reduced FVC), and the restriction could not be verified because body plethysmography was not done in this study. Such participants were classified as subjects with PRISm. Finally, subjects who retained FEV1/FVC below LLN after bronchodilation were considered obstructive.

### 2.4 Statistical analysis

The primary outcome of interest was the PRISm prevalence measured as the number of subjects with PRISm in the entire sample. In this study, we first tested all variables for normality with the Shapiro–Wilk test and found that the majority of them were non-normally distributed. Therefore, we only used and reported non-parametric tests for comparisons. Data across the manuscript are presented as medians with their corresponding interquartile ranges (IQR), whereas binary variables representing the prevalence of all included variables are shown as a percentage of the group. Univariate comparisons of the former included the Mann–Whitney U-test for age, BMI, CCI, CAT, mMRC, and all lung function parameters reported in this analysis. Similar comparisons for the binary variables included the χ2 test from contingency tables.

The selected predictors were first tested for PRISm in the univariate comparisons. VGDF was tested as a composite variable and then for each domain. *p*-values for such tests of occupational exposure domains were corrected using the Benjamini–Hochberg procedure because eleven exposures tested simultaneously assumed some multiplicity. The variables which were significantly associated with PRISm were then treated as predictors of PRISm in a logistic regression, firstly in crude models. In addition to the univariate comparison of PRISm with non-PRISm, we also ran another analysis comparing PRISm with normal subjects. Variables with some significant association (*p* < 0.05) with the outcome from such a test were then included in the adjusted model. Additionally, the model also included VGDF ever-exposure, pack-years of smoking, and the city where data were collected. To test whether the non-significant association of VGDF exposure with PRISm was consistent, we retested this exposure in the model, in which we included VGDF exposure in years (per 10 years of exposure). Both crude and adjusted models produced odds ratios (ORs) with their corresponding 95% confidence intervals (CIs) for each predictor. Interactions between variables were also checked in the regression models and, should there be any, stratified analyses were applied. In all procedures, *p*-values below 0.05 were considered statistically significant, and all tests were completed in NCSS2025 (Utah, USA).

## 3 Results

In total, there were 2,275 participants, including 54% of men (Table 1), and more than one-third of them had a university degree or higher. On average, subjects were overweight (median BMI 25.6 kg/m^2^). One-fourth were daily cigarette smokers at the time of examination, but only 4% reported regular use of electronic cigarettes. Furthermore, 60% were classified as exposed to VGDF using JEMs. Only 26% were engaged in regular physical activity three times a week or more. When men were compared with women, we found differences not only in basic demographics but also in socioeconomic status, as significantly more women had higher levels of education and thus were exposed to VGDF less often in their lifetimes. Smoking status was also affected by age, and far fewer women reported current daily smoking. Sex also affected the level of regular physical activity (more men reported regular exercise) and the structure of occupational exposures. Thus, men were more exposed to gas, dust, fume, mineral dust, diesel, fiber, and metal. In contrast, women were more exposed to vapor, bio-dust, mist, and asbestos, reflecting their greater likelihood of employment in medicine.

**Table 1:** Demographic, exposure, and lung function portraits of the sample.

Variable	Overall	Men	Women
*N* (%)	2,275 (100%)	1,229 (54)	1,046 (46)
Age, years*	41 (29; 56)	36 (25; 50)	47.5 (34; 60)
BMI, kg/m^2^*	25.6 (22.5; 29.4)	25.4 (22.5; 28.7)	25.8 (22.7; 30.8)
University degree or higher, *N* (%)*	878 (39)	379 (31)	499 (48)
Cigarette smoking status*			
Never-smoker	1,341 (59)	463 (38)	878 (84)
Former smoker	359 (16)	285 (23)	74 (7)
Daily smoker	546 (24)	464 (38)	82 (8)
Occasional smoker	29 (1)	17 (1)	12 (1)
Electronic cigarette use, *N* (%)*	82 (4)	45 (4)	37 (4)
Regular exercise, *N* (%)*	583 (26)	415 (34)	168 (16)
Lifetime exposure to VGDF, *N* (%)*	1,367 (60)	835 (68)	532 (51)
Exposure to vapor, *N* (%)*	695 (31)	336 (27)	359 (34)
Exposure to gas, *N* (%)*	819 (36)	518 (42)	301 (29)
Exposure to dust, *N* (%)*	1,169 (51)	664 (54)	505 (48)
Exposure to fume, *N* (%)*	734 (32)	607 (49)	127 (12)
Exposure to biodust, *N* (%)*	639 (28)	228 (19)	411 (39)
Exposure to mineral dust, *N* (%)*	844 (37)	594 (48)	250 (24)
Exposure to diesel, *N* (%)*	544 (24)	452 (37)	92 (9)
Exposure to fiber, *N* (%)*	486 (21)	404 (33)	82 (8)
Exposure to mist, *N* (%)*	441 (19)	185 (15)	256 (24)
Exposure to asbestos, *N* (%)*	701 (31)	292 (24)	409 (39)
Exposure to metal, *N* (%)*	203 (9)	183 (15)	20 (2)
FEV_1_, z-score*	−0.63 (−1.34; 0.06)	−0.56 (1.24; 0.09)	−0.71 (−1.43; 0)
FVC, z-score*	−0.73 (−1.38; −0.07)	−0.68 (−1.32; 0)	−0.79 (−1.41; −0.16)
FEV_1_/FVC, z-score	0.12 (−0.49; 0.68)	0.12 (−0.5; 0.72)	0.13 (−0.49; 0.66)
PRISm, *N* (%)*	273 (12)	129 (10)	144 (14)

PRISm, preserved ratio impaired spirometry; BMI, body mass index; VGDF, vapor, gas, dust and fume; FEV_1_, forced expiratory volume in 1 s; FVC, forced vital capacity; *p < 0.05 in the univariate comparisons using either *χ*^2^ test for binary data or Mann–Whitney U-test for continuous variables.

Quality of spirometry in this study was very high. More than 90% of tests were scored A or B either for ΔFVC or ΔFEV1, or both. The remaining tests were scored C, and all tests were included in the analysis. Overall, exhaled volumes were much lower in women (Table 1, Mann–Whitney *p*-values for FEV1 and FVC z-scores below 0.01), but FEV1/FVC was similar. We classified 12% (95% CI 10.7; 13.3) of subjects as having PRISm, with a significantly higher prevalence in women (14, 95% CI 11.7; 15.6) compared to men (10, 95% CI 8.8; 12.2). Among participants with PRISm, 200 subjects had FVC below LLN. The non-PRISm group comprised subjects classified as subjects with normal spirometry (N = 1882, 94%) and obstructive subjects (*N* = 120, 6%). We then compared participants with PRISm with those with no PRISm and found that sex, BMI, regular exercise, CCI, CAT, and mMRC were associated with the outcome, but not age, education, smoking, or lifetime exposure to VGDF (Table 2). Since the comparison group also included obstructive subjects, we then compared the distribution of variables between the PRISm group and subjects with normal spirometry only. In such univariate comparisons, age was also associated with the outcome in addition to significant predictors in Table 2 (sex, BMI, regular exercise, CCI, CAT, and mMRC). Notably, female sex, age, and BMI increased the likelihood of PRISm, but regular exercise was protective (*p* < 0.05). Furthermore, CCI and mMRC median scores did not differ between these groups, but the score difference was statistically significant using the Mann–Whitney U-test. In PRISm subjects, all tested clinical variables, including CCI, CAT, and mMRC, were greater (*p* < 0.05).

**Table 2:** Selected demographic, occupational and clinical predictors of PRISM in the univariate comparisons.

Variable	PRISm	Non-PRISm
*N* (%)	273 (12)	2,002 (88)
Women, *N* (%)*	144 (53)	902 (45)
Age, years	45 (30; 57)	41 (28; 56)
BMI, kg/m^2^*	27.5 (23.4; 31.2)	25.4 (22.5; 29.2)
University degree or higher, *N* (%)	101 (37)	777 (39)
Never-smokers, *N* (%)	170 (62)	1,171 (58)
Electronic cigarette users, *N* (%)	6 (2)	76 (4)
Regularly exercising subjects, *N* (%)*	55 (20)	528 (26)
Lifetime exposure to VGDF, *N* (%)	166 (61)	1,201 (60)
Exposure to vapor, *N* (%)	78 (29)	617 (31)
Exposure to gas, *N* (%)	97 (36)	722 (36)
Exposure to dust, *N* (%)	141 (52)	1,028 (51)
Exposure to fume, *N* (%)	89 (33)	645 (32)
Exposure to biodust, *N* (%)	87 (32)	552 (28)
Exposure to mineral dust, *N* (%)	99 (36)	745 (37)
Exposure to diesel, *N* (%)	64 (23)	480 (24)
Exposure to fiber, *N* (%)	58 (21)	428 (21)
Exposure to mist, *N* (%)	55 (20)	386 (19)
Exposure to asbestos, *N* (%)	88 (32)	613 (31)
Exposure to metal, *N* (%)	25 (9)	178 (9)
CCI*	1 (0; 3)	1 (0; 2)
CAT*	1 (0; 7)	0 (0; 4)
mMRC*	0 (0; 0)	0 (0; 0)

PRISm, preserved ratio impaired spirometry; BMI, body mass index; CCI, Charlson comorbidity index; CAT, chronic obstructive pulmonary disease assessment test; mMRC, modified Medical Research Council; VGDF, vapor, gas, dust, and fume; *p < 0.05 in the univariate comparisons using either *χ*^2^ test for binary data or Mann–Whitney U-test for continuous variables; *p*-values for specific exposures (*N* = 11) are corrected for multiplicity using the Benjamini–Hochberg procedure.

Despite the non-significant VGDF effect, we retained this variable in the adjusted model as a binary variable of ever-exposure, as well as pack-years of smoking as a continuous variable. In the multivariate model including sex, age, BMI, regular exercise, ever-VGDF exposure, pack-years of smoking, CCI, and even the city, only BMI, CCI, and the city retained a significant association with PRISm (Table 3). Another model with VGDF duration in years as opposed to ever-exposure consistently showed no effect of such exposure [crude OR for every 10 years of exposure 1.11 (95% CI 0.99; 1.24); adjusted OR 1.06 (95% CI 0.93; 1.20)]. In a sensitivity analysis in only 73 PRISm patients with normal FVC, the association with VGDF exposure was consistently non-significant, whether the exposure was expressed as ever-exposure [OR 0.86 (95% CI 0.52; 1.42)] or lifetime exposure in years [OR 1.00 (95% CI 0.79; 1.25)]. However, we found some interaction of exposure to fume with regular exercise, as well as sex with BMI and even with regular exercise. Therefore, we retested the effect of exposure to fume separately among exercising and non-exercising participants, but even in these stratified analyses, exposure to fume failed to exhibit an association with PRISm in the adjusted models.

**Table 3:** Logistic regression analyses of selected variables with PRISm showing ORs and their 95% CIs.

Variable	Crude	Adjusted
Female sex	1.35 (1.05; 1.74)	1.22 (0.90; 1.66)
Age	1.01 (1.00; 1.02)	0.98 (0.96; 1.00)
BMI	1.05 (1.03; 1.07)	1.05 (1.02; 1.07)
VGDF	1.07 (0.82; 1.38)	1.16 (0.88; 1.53)
Pack-years of smoking	1.01 (1.00; 1.02)	1.01 (1.00; 1.03)
Exercise	0.66 (0.48; 0.90)	0.75 (0.54; 1.05)
City of residence (Aktobe vs. other)	1.15 (0.89; 1.49)	1.46 (1.09; 1.96)
CCI	1.15 (1.06; 1.24)	1.28 (1.07; 1.54)

PRISm, preserved ratio impaired spirometry; ORs, odds ratios; CIs, confidence intervals; Variables were tested in the PRISm group compared to subjects with normal spirometry (*N* = 1,882); BMI, body mass index; CCI, Charlson comorbidity index; VGDF, vapor, gas, dust, and fume; Adjusted models are fully adjusted for each shown variable.

## 4 Discussion

This population-based study in Kazakhstan aimed to test the association between occupational exposures classified with JEM and PRISm. The rationale for this hypothesis was based on the findings of some previous studies, in which PRISm was believed to progress to obstruction, and obstruction, in turn, was strongly related to airborne occupational exposures. In our multivariate analyses, we could not confirm our initial hypothesis and found no evidence of such association. None of the tested exposures, including vapor, gas, dust, fume, and others, demonstrated a significant association with PRISm. Nevertheless, we confirmed some earlier reports indicating a strong association between PRISm and BMI, as well as with comorbidities, providing more external validity to our study methodology. In addition, we also found that the prevalence of PRISm in the study population was somewhat similar to that in most other epidemiological studies elsewhere and remained close to 10–13%.

At present, PRISm is a widely studied phenomenon with unclear implications for clinical management. Depending on the definition, approach to classification, setting, study design, and the criteria, its prevalence may vary widely, staying within the range of 7–13% in the majority of cases (2, 3, 5, 9, 18–20), but selected studies reported the prevalence reaching 20% (4). There exists some geographical disparity in the reported PRISm prevalence, but it is still unclear whether these inconsistent findings reflect a true contrast in epidemiology or result from variations in methodologies. Because an array of PRISm determinants, including obesity as an example, can vary dramatically across regions, PRISm may also exhibit inconsistent prevalence around the globe. Our estimated prevalence of 12%, with a higher prevalence in women compared to men, is consistent with the majority of published studies elsewhere and likely reflects the expected prevalence of PRISm determinants in the general population in Kazakhstan. In this 12% of the population, obviously some clinical investigation may be needed once PRISm is diagnosed, because this spirometric phenomenon may help identify other conditions which may remain unnoticed otherwise.

The clinical importance of PRISm should be further discussed, given that PRISm may transition to COPD, as has been shown in many previous studies (9, 21). PRISm and COPD have some overlapping predictors and risk factors, including cigarette smoking (4, 8, 22); therefore, hypothesizing that PRISm may be viewed as a stage to COPD is plausible and confirmed in some other studies. The mechanism by which PRISm transitions to COPD has yet to be verified, but decline in accelerated lung function induced by cigarette smoking can explain why approximately one-third of subjects with PRISm will develop COPD (3). Even if the exact mechanisms of PRISm and COPD may be different, patients with PRISm should still gain clinical attention because they may suffer from compromised lung function, without showing clinical manifestations at earlier stages. Therefore, identifying PRISm patients earlier can help plan targeted case finding for COPD and address the mitigation of risk factors, such as smoking. Although our study did not aim to test the effect of such interventions, longitudinal evidence from other sources suggests that these interventions are necessary. Smoking cessation is widely beneficial not only for smokers but also for those exposed to environmental tobacco smoke (23). However, addressing this associated factor in the earlier stages, such as in PRISm, remains a topic of scientific debate due to scarce evidence. Overall, access to spirometry may still be limited in many settings, thus making early PRISm detection challenging.

This study failed to identify the association between PRISm and airborne chemical exposures in the workplace, which were classified with JEM. Occupation has traditionally been overlooked in studies of PRISm, creating a knowledge gap since such a link could theoretically be plausible. While sporadic reports have found an association only for indium (24), workers in the industry can be exposed to a very wide array of metals and other elements (25, 26); however, no conclusive evidence on the association between occupation and PRISm can be found in the biomedical literature. Despite some plausibility, the non-significant association between occupational exposures and PRISm in our study might have resulted from comparator definition, exposure and outcome misclassification, imprecision in our measurements, or even residual confounding. Further studies with better exposure classification may provide more insights into the occupational risk factors associated with PRISm. In Kazakhstan, local environmental factors are also relevant to the exposures of interest. In particular, ambient air pollution and the associated indoor air pollution are urgent public health concerns (27–29). Ambient air pollution during the heating season in cities may be alarming, and the levels are worrisome, whereas the mechanism of action increasing the odds of PRISm may be quite similar to the occupational airborne-associated factors.

In the current study, we confirmed the findings of earlier studies that there existed an association between comorbidities and PRISm. Other larger studies have consistently demonstrated that diabetes, hypertension, coronary artery disease, heart failure, and even kidney disease may be linked to PRISm (4, 6–8, 30, 31). We used a combined index, CCI, in our analysis as an alternative to selected diagnoses. One has to bear in mind that self-reported chronic diagnoses are often prone to recall bias, and such poor recall with regard to chronic conditions is very prevalent in the Kazakhstan population. Thus, our previous study of COPD found that only 24% of participants with COPD via spirometry in the study had been informed of their condition beforehand (11).

Factors associated with PRISm progression are a subject of scientific debate. These factors are expected to be somewhat similar to the risk factors for this condition, and cigarette smoking is the most studied factor of PRISm progression (32). Other progression factors, such as the PRISm asthma phenotype and some others, are under study and require deeper investigation (33). Future studies should further explore other progression factors of interest, which may include not only intrinsic determinants but also environmental conditions.

The ulticenter design and the use of z-scores as an alternative to percent predicted values for spirometric volumes can be considered the strengths of this presentation. The limitations of our study include incomplete clinical data and some potential exposure misclassification related to clinical conditions. All of these were self-reported with no clinical confirmation, making some non-differential misclassification possible. Secondly, this study employed a cross-sectional design, which made it impossible to confirm temporality and the direction of association, and this could be extrapolated to all associated factors in the study. In addition, cross-sectional studies do not allow researchers to ascertain mortality, which is an important clinical outcome for scientific inquiry regarding PRISm. Thirdly, since this is a population-based approach, we could not supplement the diagnosis with radiological examinations, such as chest X-rays or computed tomography (CT) scans. Finally, there is no strict diagnostic approach for assessing bronchodilation in epidemiological studies when PRISm is considered. Recent studies address the issue of phenotypes and subtypes of PRISm, raising the question of whether pseudo- and true restriction should be ruled out in individuals having an initial PRISm spirometric pattern (34). At present, no uniform classification exists, and distinct criteria to discriminate between PRISm and a restrictive pattern, as well as the need for bronchodilation in all subjects, are criteria.

Our results yield clear implications for public health practices, mostly related to prevention. PRISm is a prevalent condition that clinicians should recognize, as they often only address spirometric abnormalities when they have progressed to COPD. This delay reflects a window of lost opportunities because communicating the need to reduce weight, cease smoking, and control comorbidities could prevent a large fraction of future COPD cases. More high-quality studies quantifying the effects of such interventions are important because evidence is needed on whether the efforts and resources dedicated to working with PRISm patients provide a substantial return on investment. However, all these prevention plans may face the risk of limited availability of spirometry and overall low quality. Therefore, public health decision-makers should propose a comprehensive national plan to ease access to high-quality spirometry in Kazakhstan and other Central Asian countries.

In conclusion, this first study on the prevalence of PRISm and the association of selected determinants with it in our study population in Kazakhstan showed that its prevalence may be approximately 12%, similar to that in many other countries around the world. Lifetime occupational exposure to airborne chemical agents, such as vapor, gas, dust,and fume, defined using JEMs, yielded a non-significant association with PRISm in our study, but high BMI and comorbidity remained significantly associated with PRISm in our study population.

## Data Availability

The raw data supporting the conclusions of this article will be made available by the authors, without undue reservation.
